# Bone Tissue and the Nervous System: What Do They Have in Common?

**DOI:** 10.3390/cells12010051

**Published:** 2022-12-22

**Authors:** Arianna Minoia, Luca Dalle Carbonare, Jens Christian Schwamborn, Silvia Bolognin, Maria Teresa Valenti

**Affiliations:** 1 Department of Medicine, University of Verona, 37100 Verona, Italy; 2 Luxembourg Centre for Systems Biomedicine (LCSB), Developmental and Cellular Biology, University of Luxembourg, 4365 Belvaux, Luxembourg; 3 Department of Neurosciences, Biomedicine and Movement Sciences, University of Verona, 37100 Verona, Italy

**Keywords:** stem cells, bone, neurogenesis, differentiation

## Abstract

Degenerative diseases affecting bone tissues and the brain represent important problems with high socio-economic impact. Certain bone diseases, such as osteoporosis, are considered risk factors for the progression of neurological disorders. Often, patients with neurodegenerative diseases have bone fractures or reduced mobility linked to osteoarthritis. The bone is a dynamic tissue involved not only in movement but also in the maintenance of mineral metabolism. Bone is also associated with the generation of both hematopoietic stem cells (HSCs), and thus the generation of the immune system, and mesenchymal stem cells (MSCs). Bone marrow is a lymphoid organ and contains MSCs and HSCs, both of which are involved in brain health via the production of cytokines with endocrine functions. Hence, it seems clear that bone is involved in the regulation of the neuronal system and vice versa. This review summarizes the recent knowledge on the interactions between the nervous system and bone and highlights the importance of the interaction between nerve and bone cells. In addition, experimental models that study the interaction between nerve and skeletal cells are discussed, and innovative models are suggested to better evaluate the molecular interactions between these two cell types.

## 1. Introduction

Few studies have thoroughly analyzed the connections between the nervous system and bone. However, there is a relationship between these two systems, and some pathologies indicate the association between bone tissues and the nervous system. If we consider the two systems from an evolutionary perspective, it is apparent that the development of one influenced the development of the other. The modern human differs from the Neanderthal human not so much in the size of the brain as in the shape of the head [1]. The globularity of the modern human head is the result of a specific differentiation process after birth, at the stage where the brain determines the shape of the skull [2]. Therefore, it has been suggested that the globular shape of the skull of modern humans is not limited to a morphological change but is associated with neurofunctional processes [3]. 

Alterations in the balance of bone metabolism lead to a greater risk of fractures and an increase in osteoporosis [4]. The major hormonal variables that control bone metabolism are those that react to simultaneously perceived paracrine, autocrine and mechanical inputs [5]. Peripheral nerves regulate bone remodeling and recently it was demonstrated that sensory/motor nerve injury causes osteoporosis [6]. In addition, there is a relationship between osteoporosis and psychological stress, regulated by the hypothalamic–pituitary–adrenal (HPA) axis, glucocorticoid levels and a reduced response to factors that increase bone mass [7,8]. Chronic stress, through the production of inflammatory factors, promotes osteoclast differentiation as well as osteoblastic apoptosis [7], and it has been reported that anxiety levels can be considered a prognostic risk of fractures in postmenopausal women [8]. Importantly, although psychological stress and osteoporosis are distinct conditions, some factors such as glucocorticoids, catecholamines and insulin-like growth factors are involved in both alterations [9]. Osteoporotic fractures can be associated with spinal cord injuries. This lesion causes severe disability, compromising quality of life. After a spinal cord injury, the bone loss observed can be as high as 40% and the fractures can have devastating consequences [10]. Disuse following a spinal cord injury promotes increased levels of sclerostin, produced by osteocytes, reducing bone formation and inducing bone resorption through osteoclastic activation [11].

It has been reported that Alzheimer’s disease (AD) patients show lower bone mineral density and these patients often have fractures [12]. As osteoporosis has been shown to correlate with cognitive impairment, this condition is considered a risk factor for the development of AD [13]. In addition, Parkinson’s disease (PD) patients can be affected by Pisa syndrome due to trunk scoliosis, leading to further complications of disability [14]. Patients with AD and PD suffer from bone problems and osteoarthritis, and they have limited movement. 

In addition, osteoporosis or skeletal abnormalities produce molecules capable of promoting neurodegenerative progression [15]. In a previous meta-analysis, the authors found that PD patients have a significantly increased risk of osteoporosis and osteopenia, and that female patients are more severely affected than male patients [16]. This gender difference in osteoporosis can be explained by the important role of endocrine and nutritional factors. From another point of view, it is known that bone mineral density is negatively affected by several neurotoxic metals, such as cadmium, lead, aluminum and arsenic, and these metals are implicated as well in neurodegenerative disorders [17].

Another important relationship between bone disease and neurodegenerative diseases is vitamin D levels. Hypovitaminosis D is associated with decreased bone mineral density and an increased risk of fractures [18], and accumulating evidence shows that vitamin D is essential for proper brain development, maturation and functions and neural network development [19]. Vitamin D deficiency seems to be related to various neurological conditions, such as MS, PD and AD, and vitamin D supplements resulted in no significant benefits for improving motor function for patients with PD [19,20].

In addition, vitamin D deficiency is associated with an increased risk of falls [21], worsening these events in patients with neurodegenerative diseases and further increasing the risk of fractures.

Cognitive alterations and motor disabilities are present in CDKL5 deficiency disorder (CDD). Patients with this disorder, a rare condition of X-linked impaired neurogenesis, have seizures and cognitive impairment and are characterized by microcephaly and scoliosis [22,23]. Interestingly, the Cdkl5 mutant zebrafish has been shown to be characterized by skeletal and neuronal disorders [24]. It has been suggested that CDKL5 plays an important role in bone metabolism and hypermethylation has been observed in osteoporotic patients with this gene [25].

On the basis of these reported findings and to understand the relationship between the two systems, it seems appropriate to evaluate the biology of the cells from which these systems derive and, in particular, the molecular crosstalk occurring between bone and nervous cells both at the physiological and pathological levels. The aim of this review is, therefore, to describe the “intersection” between the bone and the nervous system through the knowledge of related cellular and molecular systems. Finally, the intent of this review is also to provide suggestions and experimental models to identify therapeutic approaches/intervention tools to counteract some of the degenerative diseases that currently affect many subjects.

## 2. Search Strategy

Studies on the bone and nervous system were selected by consulting public databases. In particular, we identified 397 full articles from the following databases: PubMed, Web of Science and Scopus. The following keywords were used to search the titles and abstracts in all databases: bone, osteoblasts, stem cells, brain, nervous, diseases, molecule delivery in vitro experiments and in vivo experiments, with the Boolean operators (AND/OR/NOT).

Then, we removed duplicates and chose the article abstracts based on their agreement with our review topic. We removed several reviews and papers that were not recent. Thus, 226 papers were included in this review and are cited in the references.

## 3. Bone Marrow Mesenchymal Stem Cells (BM-MSCs)

The bone is a dynamic tissue that consists mainly of skeletal cells and bone marrow (BM) and has various roles, such as mineral metabolism and the generation of immune cells, hematopoietic stem cells and mesenchymal stem cells.

It has been reported that non-hematopoietic stem cells present in BM not only provide support to the microenvironment of hematopoietic stem cells, but also, thanks to their multipotency, are capable of differentiating into osteoblasts, chondroblasts and adipocytes [26].

MSCs represent a small population (from −0.001 to 0.01%) of the total nucleated cells. MSCs or MSC-like adult stem cells could be found and isolated from different mature tissues, such as adipose tissue, umbilical cord, amniotic fluid and peripheral blood [27]. The lineage commitment is promoted by specific molecular regulators of lineage. Chondrogenesis is regulated by interconnected molecular and cellular processes during embryogenesis. This is a multi-step process involving the recruitment, migration, proliferation and differentiation of mesenchymal cells. Furthermore, cellular interactions with the surrounding matrix and growth factors that modulate several transcription pathways regulate this process. The activation of cellular signaling, such as the Hedgehog signaling or Wnt signaling pathways, has been shown to play crucial roles in the development of cartilage tissue [28]. It has been reported that the Wnt/β-catenin pathway activates the expression of RUNX2, the master gene of osteogenic differentiation [29]. Yap and Taz, interacting with β-catenin, promote osteogenesis and inhibit chondrogenesis in neural crest cells [30]. The RUNX2 gene is not only the master gene of osteogenesis but is also associated with the regulation of neuronal processes. The RUNX2 gene is implicated in cleidocranial dysplasia [31] and acromegaly [32]; it controls the closure of cranial sutures [33] and is involved in the globularization of the skull [34,35]. In addition, RUNX2 participates in the development of the hippocampus [36,37] and the thalamus [38]. Mutations in RUNX2 are associated with mental disorders [39,40]. Therefore, RUNX2, whose expression plays an important role in the brain development (thalamus, hypothalamus and hippocampus) [38,41] is considered a candidate gene for serious mental illnesses, such as schizophrenia and bipolarity [37,39,40,42]. Moreover, RUNX2 transcriptionally activates osteocalcin and osteopontin [43], which, in addition to being important proteins of the bone matrix, are also involved in the organization of the brain [44].

Microglia, which are brain-specific macrophages, are another group of BM-derived cells that are in charge of the immunological defense of the brain via innate immunity mechanisms [45]. Some studies suggest that BM-derived hematopoietic cells infiltrate the brain and are subsequently able to differentiate into microglia, acquiring the ability to enter the CNS while keeping the blood–brain barrier (BBB) intact, thus colonizing the CNS especially in certain neurodegenerative diseases [46].

A fundamental role in this part is played by CCR2 (C-C chemokine receptor type 2) and CCR5 (C-C chemokine receptor type 5), chemokine receptors involved in the migration of microglial cells that have been shown to guide microglia derived from BM through the BBB, causing their accumulation in the brain parenchyma [45]. A reduced expression of CCR2 also leads to a decrease in BM-derived microglial cells within the brain and an increase in amyloid-β peptide levels [47].

## 4. Crosstalk between Bone and Neural Cells

Many neurotransmitters affect the metabolism of bone cells, and there is evidence that the central nervous system, which controls bone tissue directly via efferent neural connections, plays a significant role in maintaining the homeostasis of bone metabolism. [48]. In particular, the skeleton is innervated by a complex peripheral nervous system. The peripheral nervous system, by infiltrating the bones, regulates skeletal homeostasis by controlling bone metabolism and stem cell activity and secreting neurofactors [49].

Despite the observed relationship between the bone and neuronal system, few studies have investigated the presence of pathways common to bone and neuronal cells. One of the main and important pathways that links bone metabolism and the brain is the Wnt/β-catenin pathway. The Wnt pathway is regulated through the activation and inactivation of non-canonical and canonical signals during brain development [50].

The homeostasis of bone tissue is fundamentally regulated by the Wnt/β-catenin pathway. When Wnt is secreted, it can connect to the Frizzled and Lrp5/6 receptors, causing β-catenin to accumulate in the cytoplasm and move into the nucleus to control gene expression [51]. Additionally, it has been demonstrated that parkin, an E3 ubiquitin ligase involved in neurodegenerative diseases [52], can control the differentiation of BMSCs into osteogenic lineages via modifying β-catenin signaling and the autophagy process. In fact, the overexpression of parkin could induce β-catenin expression and the autophagy process through the expression of specific osteo-markers [53]. Leptin has been shown to be involved in modulating bone mass by acting on the hypothalamus and altering the sympathetic system. In particular, the activity of leptin induces the release of noradrenaline, which, in turn, activates the beta-adrenergic receptors expressed by osteoblasts [54,55]. It has been reported that neuromedin, through hypothalamic signals, regulates bone mass by inducing the expression of osteoblastic genes [56]. Recently, particular interest has been shown to the evaluation of the microenvironment inside the bone, in particular the neural component, and the regulatory role of the peripheral neural system on bone metabolism has been reported [57,58]. Sensory nerves are present in both cortical and trabecular bone motor nerves [58]. The intraosseous motor nerves are divided into adrenergic and cholinergic and communicate with bone cells with the aim of regulating bone metabolism through neurotransmitters. Peptidergic neurons were observed at the level of the periosteum and mineralization sites, in contact with osteoblasts, osteoclasts, stem cells and hematopoietic and endothelial cells [59]. Sympathetic nerve fibers, at the level of osteoblasts and osteoclasts, release neurotransmitters and neuropeptides capable of regulating bone cells and, thus, skeletal metabolism [60]. Norepinephrine (NE), the principal neurotransmitter of the sympathetic nervous system found in bone, inhibits bone formation. 𝛽-adrenergic receptors (β-Ars) are present in osteoblasts and osteoclasts with the function of regulating bone metabolism [61,62,63]. This regulation takes place through noradrenaline, which activates β-ARs present in osteoblasts and reduces bone formation. In particular, reduced bone formation is a consequence of the expression of RANKL and interleukins (IL)-6 and IL-11, which in turn promote osteoclast differentiation and maturation [63,64]. β-ARs can also directly regulate osteoclastogenesis by promoting the formation of reactive oxygen species [65]. In addition, the differentiation of mesenchymal cells can also be regulated by β-Ars via the cAMP/PKA pathways [58]. Sometimes, β1-ARs and β2-ARs regulate bone remodeling in the opposite way by exerting anabolic and catabolic activity, respectively [58].

The nicotinic acetylcholine receptors (nAChRs) or muscarinic acetylcholine receptors bind to the acetylcholine (ACh) that is released by cholinergic neurons (mAChRs). It has been reported that ACh regulates bone metabolism through these receptors, also expressed by bone cells [66,67,68]. Another factor associated with bone remodeling is the calcitonin-gene-related peptide (CGRP), which stimulates osteogenic differentiation by the upregulation of transcription factor-4 (ATF4) and osteocalcin and reduces osteoclastogenesis [69,70,71]. Furthermore, CGRP is involved in the bone’s ability to adapt to mechanical stresses, such as compression [72]. In addition to being crucial for the growth, survival and differentiation of nerve cells, neurotrophins, such as nerve growth factor (NGF), brain-derived neurotrophic factor (BDNF), glial-cell-line-derived neurotrophic factor (GDNF), neurotrophin 3 (NT-3) and neurotrophin 4/5 (NT 4/5) are also involved in bone metabolism and are essential for the communication between peripheral nerves and the bone [58].

Bone cells, by releasing osteokines such as osteocalcin and lipocalin-2 that are able to pass through the BBB, communicate with the brain. Osteocalcin, accumulating in the brainstem, can affects neurotransmitter synthesis as well as cellular signaling, thus influencing age-related memory loss [73]. About 50% of circulating lipocalin-2 is produced by osteoblasts. In the hippocampus, an increase in LCN2 has been observed following inflammation, and the administration of LCN2 in the CNS has also been shown to reduce food intake and body weight [74]. The fibroblast growth factor-23, produced by osteoblasts and osteocytes, has been also found in the hypothalamus, hippocampus and cortex, and an alteration of cognitive functions in the FGF23 knockout mouse has been observed [75]. RANKL, detected in the hypothalamus, controls body temperature in females [76]. In addition, it has been shown that the treatment with anti-RANKL reduces stress and depression in mice with chronic social defects [77]. Osteopontin (OPN), osteoprotegerin (OPG), Sclerostin and Dickkopf-124 are other osteokines that can impact neuronal cells [78,79,80].

In neural cells, the expression of SATB2, a DNA-binding protein involved in the development of the brain, depends on both Bone Morphogenic Proteins (BMP) and SHH [81] and downregulates HOXA2, a target gene of the language regulator FOXP2 [82,83]. HOX2A plays an important role in both brain and bone generation [84,85], and it is an important gene for skeletal morphology [83,86,87]. Interestingly, the activation of HOXA2 in the neural crest reduces the expression of BMP inhibitors, causing craniofacial and also cerebral alterations [88]. On the other hand, FOXP2 is involved in both bone formation and neuronal stem cell commitment during corticogenesis [89,90]. HES1, functionally related to RUNX2, plays an important role during neurogenesis by regulating the Slit/Robo pathway [91] as well by affecting GABAergic and dopaminergic neuron formation [92]. In particular, Hes1 silencing induces GABAergic neuron differentiation in BM-MSCs [92] and is involved in osteoarthritis diseases [93].

## 5. Experimental Models for the Study of Skeletal and Neuronal Cells 

### 5.1. In Vitro Models

In order to analyze and study the crosstalk between neuronal cells and bone metabolism, both in vitro and animal models have been used [58,94]. Regarding the study of bone metabolism, it was found that the use of in vitro models in 2D cell cultures are effective for functional and gene expression studies as well as for protein analysis [95] (Table 1). However, to fully capture the complexity of human tissues and the interplay between several cell lines, more sophisticated 3D models are necessary. Numerous papers have addressed the differences between cells cultured in 2D vs 3D models, showing that cell morphology, proliferation and differentiation are closer to the physiological situation in 3D [96,97]. Three-dimensional models are useful for detailed research of stem cell behavior, drug development, disease modeling and genetic screening. In fact, 3D models can greatly multiply tissue-specific stem cells and their differentiated cells from incredibly small amounts of starting material [98]. In the AD context, it has been shown that iPSC-derived neurons in 3D can be used for phenotypic assessment [99]. It has been demonstrated that the 3D framework provided by Matrigel, as well as other hydrogels including collagen and alginate gels, expedites the formation of neural networks. More crucially, the matrix’s support enables vertical development, which is entirely impossible in 2D cultures and leads to unfavorable apical–basal polarity [100].

Additionally, the 3D environment has the ability to elicit mechanical cues that can be translated into biochemical signals that are underrepresented in traditional 2D cultures [101]. It has also been shown that 3D set-ups can be compatible with multiplexing and automated screening procedures [102]. Furthermore, it has been shown that the use of neural progenitor cells with midbrain floor plate identity can be a source for the development of midbrain specific organoids. These midbrain organoids can be used to study neurodegenerative diseases such as PD. As an example, patient-specific organoids with a mutation in the PD-associated gene LRRK2 recapitulate key pathological processes, which are also seen in the actual patient’s brain [103].

Bone organoids are 3D self-renewing and self-organized micro-bone tissues with biomimetic spatial features [100]. They are composed of directionally differentiated stem cells, such as bone stem cells and embryonic stem cells, or progenitor cells, such as osteoblast and/or osteoclast. Bone organoids need biocompatible materials, such as Matrigel or alternative synthesized hydrogels, applied as a support to the self-organization of the bone organoids [104]. In contrast to other 3D cell culture models (such as spheroids), bone organoids develop from tissues specific to humans and self-organize into tissues resembling organs [105].

Conversely, spheroids are densely packed cell aggregates with irregular distributions. Due to inappropriate cell types and excessive human intervention, typical bone tissue creation techniques still fall short of accurately simulating the physiological microenvironment [106].

The osteoblast/osteocyte population produced from ESCs (embryonic stem cells) exhibits excellent potential to produce bone organoids. It has been demonstrated that 3D culture conditions gradually improve the stemness, proliferation and differentiation of MSCs and that anti-inflammatory and anti-apoptotic capability also occurred [107]. As typical ECM-produced materials, Matrigel and tissue-specific extracellular matrix have been shown to facilitate successful organoid growth [108].

Matrigel, which contributes to the ECM’s chemistry, offers plentiful collagen supports and physiological properties that are biocompatible for the development of organoids [108]. However, Matrigel is expensive; it has an unclear chemical makeup and the batch-to-batch variability leads to reproducibility issues. Biomaterials, both natural and artificial, have been used to increase the reproducibility of the experiments [109]. Thus, the organoids have been cultured using collagen, gelatin, alginate, fibrin and hyaluronic acid (HA). The two earliest synthetic polymers that are still often utilized in organoid research are polyethylene glycol (PEG) and polyisocyanopeptide (PIC) [110,111,112,113].

### 5.2. In Vivo Models

Zebrafish and mouse are generally used as in vivo models for studying degenerative diseases. The main advantage of using zebrafish as a model for brain–bone crosstalk is the fact that zebrafish and mammals share many skeletal development and biological phenomena as well as overall inventory of bone types [114]. As in the case of mammals, teleosts undergo skeletal histogenesis, which involves the differentiation of mesenchymal stem cells into chondroblasts and osteoblasts that produce the collagen extracellular matrix [115]. In both mammals and fish, skeletal cells differentiate through a complex interplay between intracellular molecular pathways and chemicals that regulate the timing, position and process [116]. Additionally, Parkin KO results in a moderate loss (20%) of dopaminergic neurons and decreased mitochondrial complex I activity in the zebrafish model [117]. In fact, zebrafish is also used as an experimental model for PD, and Parkin, PINK1 and LRKK2 are examples of related protein homologs found in zebrafish. Although fish have three synuclein genes, no homologs of human synuclein have been found [118].

Furthermore, mouse models are frequently used to study age-related diseases, such as bone disorder and neurodegenerative diseases [119]. The results of research conducted using murine models frequently challenge conventional wisdom regarding normal and pathological osseous remodeling. To discriminate among outcomes that are therapeutically meaningful, one must fully comprehend the distinctions between the physiology of human and murine bones. The evidence to date suggests that the characteristics and timing of osseous loss related to age in mice are quite similar to those of human beings [120]. Mouse models clearly demonstrate a rise in cortical porosity with age, which may provide information on the state of human beings [120]. For neurodegenerative pathologies, in particular for PD, mouse models have been extensively employed because the genetic alteration of these animals is relatively simple and common. The lifespan of mice, which is only two years, makes them a poor model for diseases such as PD, which can take five to six decades or longer to manifest in humans [121]. Therefore, with autosomal dominant disorders, the overall treatment is on self-expression in an effort to shorten the time that the disease takes to appear in mice. Results in transgenic models have been validated using human post-mortem tissue [122]. Even though post-mortem analyses of humans typically focus on diseases at an advanced stage, the early signs of disease persist and are the gold standard for evaluating how well animal models can mimic actual pathogens [121]. To understand the first anomalies in the DA nigrostriatal system that have been discovered as result of these mutations, it may be helpful to study the current knockdown parkin, PINK1 and DJ-1 [123]. Similarly, the current transgenic LRRK2 models may be very helpful for researching the developmental disorders in the DA nigrostriatal system. Some transgenic α-synuclein animal models also have increasing sensory abnormalities caused by dopaminergic dysfunction [124]. Therefore, finding a model organism capable of satisfying the needs necessary to investigate the crosstalk between brain and bone in the best possible way is a challenge. Future therapeutic strategies must consider the intensive and dynamic brain–bone interaction as well as the genetic and neuropsychological comorbidities of the affected patients. A strategy that takes this into account could be the possibility of studying bone–brain crosstalk using bone and brain organoids in on-chip approaches, which may call for extra monitoring or even tailored treatment plans. At the moment, however, these approaches are not particularly used, and these tools are used to study either the bone or the neuronal system or both simultaneously.

**Table 1:** Several in vitro and in vivo models used in skeletal and neuronal studies.

2D In Vitro Models	3D In Vitro Models	In Vivo Models
CELL CULTURE Cell culture is effective for functional and gene expression studies, as well as for protein analysis. Main cells used: SH-SY5Y, PBMS, MSCs, MLO-Y4, MLC3T3-E1, MNC and RAW264 [95,125].	Three-dimensional cell culture is useful for detailed research of stem cell behavior, drug development, disease modeling and genetic screening. Three-dimensional neural cell culture [65,99,103]; Three-dimensional bone cell culture [100,126,127]; Bone spheroids [105,128]; Neural spheroids [129,130]; On-chip organoid for brain or neural models [131,132,133,134]; On-chip organoid for bone models [135,136]	Zebrafish and mammals share many skeletal development and biological phenomena. Zebrafish for bone studies [24,114,116,137]; Zebrafish for neural studies [24,104,117,118]; Zebrafish model for bone and brain crosstalk [24,138]; Murine model for bone and brain crosstalk [72,139,140].

## 6. Bone and Neuronal Cells in Degenerative Diseases

With increasing population aging, there is also an increase in neurodegenerative diseases and osteoporosis. Many data confirm the correlation between bone and neurodegenerative diseases [13,15,141].

Alzheimer’s disease and Parkinson’s disease are among the most common forms of neurodegenerative diseases.

Alzheimer’s disease is characterized by memory loss and cognitive decline. Even if there are common neuropathological features, the molecular mechanisms involved in the genetic susceptibility and progression of AD remain to be elucidated. In AD, bone mass reduction, characterized by the increase in inflammatory markers, can be considered a risk factor [13]. Recently, it has been reported that astrocyte alterations and an imbalance in calcium levels link osteoporosis and AD [142]. Moreover, AD causes beta amyloid accumulation in the bone, promoting RANKL-induced osteoclastic activation and thus favoring bone resorption [143]. In addition, by using AD mouse models, it has been demonstrated that lipocalin-2, produced by osteoblasts and regulated by miRNA-96-5p/Foxo1, promotes AD [144]. By performing single-cell RNA sequencing analyses, the age-associated transcriptomic profile in the cells of CNS such as microglia [145] has been recently reported and it has been demonstrated that beta amyloid, a pathological biomarker of AD, is able to modulate these cells [146]. In the PSEN1 p.G378E mutated-AD mouse model, the involvement of the receptor TYROBP in disease progression has been demonstrated [147]. Interestingly, the protein TYROBP is expressed in different cells, such as microglia and osteoclasts, two cells sharing a myeloid origin. Signaling pathways, such as TREM2/TYROBP, CSF1 and CCR5, are involved in AD and osteoporosis [148,149]. Moreover, skeletal fragility due to osteoclast activation is associated with increased Aβ42 levels in the brain and has been reported in an AD mouse model [140]. During aging, increased inflammatory processes cause both bone loss (by activating osteoclast activity) and AD by increasing the levels of the protease inhibitor neuronal α2-macroglobulin involved in plaque formation [150].

PD is characterized by the degeneration of the neurons of the midbrain nigrostriatal dopaminergic (DA) system that regulate not only neuronal functions, but also bone metabolism, and the Wnt/β-catenin pathway plays a crucial role in the development of many aspects of DA neuron development [151]. PD represents the second most common age-associated neurodegenerative disorder affecting the nervous system after AD and it is clinically characterized as a movement disorder. Often, patients affected by this pathology are more prone than age-matched controls to fractures and joint and bone problems due to reduced mobility, postural instability and neurological impairment [152].

There are recent results on the interaction of Parkin and β-catenin; elevated levels of sum-active (dephosphorylated) β-catenin were found in mice lacking the protein parkin [153]. Its increase in Wnt/β-catenin signaling suggests that reduced β-catenin degradation may result in DA neurons being lost as they attempt to re-enter the cell cycle. This latter finding contrasts with the active role of Wnt/β-catenin signaling during the development of midbrain DA neurons and stem cells, suggesting that pathological adult DA neurons may require less Wnt/β-catenin signaling, while DA precursors may benefit from enhanced Wnt activation/β-catenin signaling [154,155].

**Figure 1:**
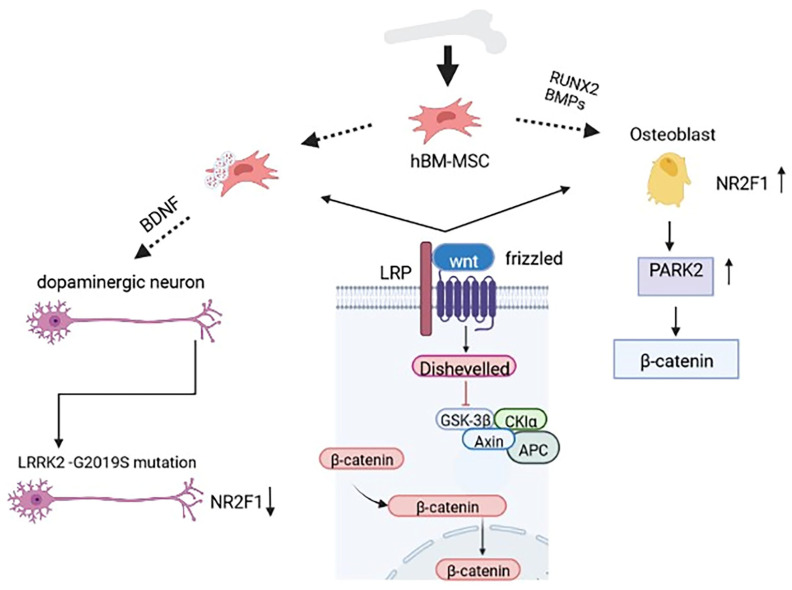
Bone morphogenetic proteins (BMPs) and the master gene of the osteoblastic commitment, RUNX2, play an important role in osteogenic differentiation. Further, the secretion of neuronal growth factors promotes neuronal commitment. Wnt/β-catenin signaling is a common pathway of these two different lines. In particular, for osteoblastic commitment, an increase in Park2 stimulates the activation of β-catenin. The expression of NR2F1 is downregulated in the DA neuron of PD patients carrying the LRRK2-G2019S mutation.

Moreover, parkin overexpression accelerates bone healing in tibial fracture model markers [53]. Additionally, parkin, which was found to be mutated in PD patients, caused impaired mitophagy with an accumulation of damaged mitochondria, causing the loss of DA neurons with age [151]. Furthermore, NR2F1, known as nuclear receptor 2 families 1 and being part of the Human Hormone Nuclear Receptor (hHNR) family, is upregulated during osteogenesis and plays a pivotal role during neurogenesis [156]. In particular, the NR2F1 transcript was recently reported to be considerably downregulated in dopaminergic neurons and midbrain organoids generated from PD patients carrying the LRRK2-G2019S mutation compared to the healthy controls [157] (Figure 1).

In addition to AD or PD, other neurodegenerative diseases are associated with bones. Multiple sclerosis and osteoporosis affect postmenopausal women and it seems that these two pathologies share pathogenetic modalities. Bisson et al. compared BMD in individuals with or without multiple sclerosis [158]. The authors observed a lower BMD and an increased prevalence of osteoporosis in individuals affected by multiple sclerosis compared to age- and sex-matched controls, suggesting that, in this population, the bone component needs to be evaluated in order to adopt a correct therapy.

Mutations in AUTS2, a gene involved in neurodevelopment as well as in several neurological disorders [159], cause cognitive impairments [160] that are often associated with skeletal anomalies [161]. Many AUTS2-associated proteins, which play an important role at the neuronal level and are associated with pathologies such as ASD and cognitive alterations, interact directly with the osteogenic master gene RUNX2 [35]. However, RUNX2 interacts with several candidate genes for autism, such as SMURF1 [162], involved in the control of axonogenesis [163,164]. SATB2, associated with ASD, cognitive disability and craniofacial alterations [165], is an important gene for osteoblastic differentiation and directly interacts with RUNX2 [166,167,168].

RUNX2, through FOXO1, interacts with DYRK1A [169], a gene located on the Down syndrome region of chromosome 21, is associated with facial and cognitive alterations [170,171] and acts as an inhibitor in the process of osteoclastogenesis [172]. In addition, DYRK1A phosphorylates SIRT1, a protein that controls neuronal and osteogenic differentiation [35]. In fact, SIRT1 upregulates and deacetylates RUNX2 and acts on β-catenin and FoxO expression in osteoblast progenitors, thereby promoting osteoblastic differentiation [35,173,174]. SIRT1 is also capable of inducing the neuronal differentiation of bone marrow mesenchymal stem cells under the activation of resveratrol [175]. AKT1, functionally related to RUNX2 [35], is involved in the regulation of the bone remodeling, a process performed by osteoblasts, cells with bone-forming activity, and osteoclasts, cells with bone resorption activity. Mutations in Akt1 and Akt2 in mice compromise bone formation [176,177]. However, AKT1 is also involved in growth-factor-induced neuronal survival [178].

Importantly, physical and cognitive alterations following traumatic brain injury (TBI) cause death or disability. TBI induces inflammation and the upregulation of leptin levels for the alteration of the blood–brain barrier or following the dysfunction of the hypothalamus–pituitary–adrenal axis. The increase in leptin levels causes the release of neurofactors, which in turn affects bone formation [179]. Additionally, heterotropic ossification is associated with TBI [180]. In particular, the reduced activity of PHD2 (prolyl hydroxylase domain proteins) due to a hypoxic environment as a consequence of TBI promotes angiogenesis; this, in turn, promotes chondrocyte hypertrophy in soft tissues, thus leading to heterotopic ossification.

## 7. Bone-Marrow-Derived Cells Influence Neurogenesis

The decline of stem cells due to aging is associated with tissue dysfunctions and reduced molecular repair.

During aging, a reduced ability of somatic stem cells to differentiate into cells fundamental for brain repair is observed. By using a mouse model, a reduced number of activated neuronal stem cells associated with aging has been observed [181]. An infiltration of immune cells into the stem cell niche is also observed during aging. The presence of immune cells, such as microglia and senescent cells, causes an inflammatory environment that surrounds the stem cell niche [182]. Thus, the modifications of the stem cell niche during aging change the spatial localization of these cells, which causes alterations between stem cells and supporting cells. In particular, the ECM produced by microglia, following inflammatory cytokine activation, causes the imbalance between oligodendrocytes and astrocytes during aging [183]. Alterations in function as well of cognition are associated with the loss of neurons and synapses in AD patients and a reduction in dopaminergic neurons in the substantia nigra in PD patients has been observed [184]. Therefore, MSCs have been considered in the attempts to counteract different neurodegenerative diseases, such as Alzheimer’s and Parkinson’s diseases, amyotrophic lateral sclerosis (ALS), spinal cord injury or traumatic brain injury.

To this purpose, different studies performed in mice reported the beneficial administration effects of stem cells [185,186,187].

It has been reported that bone marrow stem cells (BM-SCs) are able to infiltrate the brain. The migration of BM-SCs into the CNS has been observed in rodents and humans. In particular, BM hematopoietic stem cells injected in rodents are able to colonize the CNS and differentiate into non-neuronal cells as well as probably even neuronal cells [185,186,187]. In AD mouse models, MSC injection was observed to counteract cognitive decline by promoting the regeneration process by releasing extracellular vesicles and modulating neuroinflammation [188,189].

Human BM mesenchymal stem cells (BMMSCs) injected intravenously in an Alzheimer’s disease mice model were able to pass the BBB and accumulate in the hippocampus [190]. Interestingly, the authors observed that the hBM-MSC injection reduced cerebral amyloid βeta levels. In addition, several enzymes and cerebral cytokines were also modulated, probably as consequence of the hMSC presence [190]. Thus, the immunomodulatory effects of BM-MSCs could play a crucial part in the management of AD by modulating the activity of microglia and astrocytes as well as by targeting transcription factor expression and levels of cytokines involved in neuroinflammation [191].

The injection of human (h)BM-MSCs has been reported to promote the maturation of murine stem cells in the neuronal lineage via neuronal growth factor secretion [192,193]. The transplantation of hBM-MSCs into a mouse model of autism promoted an improvement in cognitive abilities, suggesting the importance of neurogenesis induced by hBM-MSC transplantation [194]. The ability of transplanted hBM-MSCs to secrete BDNF has also been evidenced in rat and mouse models of PD with cerebral ischemia [195,196,197]. hBM-MSC transplantation is also able to stimulate EGFR (epidermal growth factor receptor) expression by enhancing neurogenesis in a PD model [198]. It has been demonstrated that BM-MSC transplantation also induces the upregulation of the neuronal precursor nestin [199]. In a mouse AD model, the injection of bone-marrow-derived microglia-like cells counteracts amyloid deposition, ameliorating cognitive capacity [139].

Vesicles originated from BM/MSCs can deliver lipids and proteins as well as post-transcriptional regulators, such as non-coding RNAs, thus affecting important cellular processes [200,201]. Thus, microvesicles or exosomes produced by BM-MSCs reduce cognitive alterations due to traumatic brain injury or PD [202,203,204,205]. It has been reported that exosomes originating from MSCs can deliver molecules, such as miRNAs promoting synaptic remodeling or inhibiting neuronal apoptosis and promoting neuronal recovery in pathological models [201,202]. Exosomes produced by MSCs can deliver miR 133b in neuronal cells of a stroke rat model, thus promoting cellular regeneration in the ischemic tissue [205]. Increased levels of miR 146a are found in peripheral blood and cerebral fluids of the spine of patients with AD and amyotrophic lateral sclerosis, and it has been reported that this miR promotes remyelination by preventing NF-kB pathway activation [205,206]. Therefore, Kubota et al. reported that mir146a delivered by exosomes produced in MSCs is involved in the prevention of cognitive decline in a diabetic rat model [207]. Many studies, therefore, report the positive effect of MSCs on the protection of neuronal cells. However, the quality of stem cells is essential for carrying out effective neuroprotective functions. Senescent MSCs are associated with the development of skeletal diseases, such as osteoporosis, and neurodegenerative diseases, such as AD and PD [203].

MSCs as well as molecules produced by MSCs have been utilized to combat neurodegenerative illnesses, including AD, PD, ALS or ischemic stroke [208,209]. Huntington’s disease has been also treated with SCs [209]. In particular, the use of extracellular vesicles (EVs) from MSCs appears very promising.

Thus, the beneficial effects of MSC transplants, such as improving cognitive functions and survival, have been observed in many experimental models. The possibility of manipulating MSCs to induce their neuronal differentiation has suggested the use of MSCs to replace damaged neuronal tissues. Many researchers have performed specific protocols to differentiate hMSCs into neuronal cells that are able to produce and secrete neural factors such as dopamine or acetylcholine [210,211,212]. However, the exact molecular mechanisms underlying the role of MSCs in modulating neuronal cells are not yet fully understood. Some challenges involving the use of stem cells have not yet been resolved. In particular, stem cells can be influenced by the microenvironment, having negative consequences for correct engraftment and differentiation [5].

More studies are therefore needed, especially for traumatic brain injuries, so that stem cells can be used successfully as a therapeutic tool.

## 8. Conclusions and New Perspectives

The bone and brain are closely associated and alterations in one system are reflected in the other. In recent years, following the increase in the aging of the population, it has been observed that bone and neurodegenerative pathologies are associated. The central and peripheral neural systems are key players in the remodeling of bone. Several sensory neurotransmitters can modulate aging and osteogenic differentiation with important repercussions on skeletal pathologies. Both microglia and osteoclast cells share the same hematopoietic precursor. Pathologies, such as osteoporosis, arthrosis and bone alterations due to oncological problems, involve the crosstalk between bone and nerves. Based on the observations that highlight the importance of the crosstalk between the brain and bone, the possibility of the early treatment of bone disorders and the consequent inflammatory state appears useful to delay or reduce the severity of neurodegenerative pathologies.

Therefore, the observation of bidirectional exchanges between bone and nerves can be explored to identify new therapeutic tools. Although many studies have been performed to understand the pathogenesis of neurodegenerative diseases, many molecular mechanisms involved in the susceptibility and progression of these diseases remain to be elucidated. For this purpose, in vitro models, such as 3D models, could be useful. However, at present, experimental models are not yet perfectly complete for the study of the bone–brain crosstalk, as those currently available are aimed at studying one system or the other. In addition, at present brain on a chip is considered a dream [213].

Several studies report the beneficial effects of stem cells in neurodegenerative diseases, especially through the release of molecules in micro-vesicles or exosomes from MSCs. These cells, which are able to pass through the blood–brain barrier, could restore neuronal cell alterations due to their ability to differentiate into neuron-like cells or through fusion processes with endogenous cells. Even if stem cell therapies applied in animal models have shown successful results, many clinical applications or trials in patients with neurodegenerative diseases have not produced exciting results.

Interestingly, nanoparticles (NPs) have been proposed for the delivery of drugs or biological macromolecules for CNS application due to their potential to cross the blood–brain barrier. Recently, preliminary pharmacodynamic analyses have been performed for the treatment of AD by applying nanoparticles containing memantine (an N-methyl-D-aspartate antagonist), a molecule used to treat severe cases of AD [214].

Thus, nanoformulations, targeting tissue-specific markers, could be considered promising therapeutics tools for different diseases, including neurodegenerative disorders.
